# Toxic Encephalopathy Following Undercooked Bitter Yam Ingestion in Two Patients in Ile-Ife, Nigeria: A Case Report

**DOI:** 10.1155/crnm/8809567

**Published:** 2025-09-26

**Authors:** Uchenna C. Eke, Tajudin A. Adetunji, Ahmad A. Sanusi, Ahmed O. Idowu, Michael B. Fawale, Morenikeji A. Komolafe

**Affiliations:** ^1^Department of Medicine, Obafemi Awolowo University Teaching Hospitals Complex, Ile-Ife, Osun State, Nigeria; ^2^Department of Medicine, Obafemi Awolowo University, Ile-Ife, Osun State, Nigeria

**Keywords:** bitter yam, case report, convulsions, Ile-Ife, undercooked

## Abstract

**Introduction:** Yam is a major staple food in Nigeria and most of sub-Saharan Africa, and it is consumed in several forms. Dioscorea dumetorum is the bitter yam species found mainly in our locality. Bitter yam when undercooked can cause encephalopathy with patients presenting mainly with altered sensorium and convulsions. The cases reported here are unique because of their rarity and favourable response to supportive treatment, and they also serve to add to the existing literature on this potentially reversible cause of acute encephalopathy.

**Method:** We report two adult male patients who presented to our facility 2 months apart with multiple convulsions and unconsciousness shortly after ingestion of undercooked bitter yam. A diagnosis of generalized convulsive status epilepticus and acute repetitive seizures secondary to bitter yam poisoning was made, respectively, and they were managed successfully with intravenous phenytoin and both made a complete recovery.

**Conclusion:** These cases highlight the need for more awareness among clinicians regarding the neurological manifestations of bitter yam toxicity when poorly prepared and also for education of the public on preventive measures.

## 1. Introduction

Bitter yam is a popular staple food in Nigeria and also believed to have medicinal properties [1]. However, it contains a number of toxic components which include dioscorine and histamines [2]. Dioscorine is responsible for the central nervous and digestive system effects, while the histamines are responsible for allergic reactions such as itching [3]. Poisoning occurs when the yam is eaten raw or undercooked [2, 3]. In addition to cooking, soaking in water and subsequent drying are other effective local ways of processing bitter yams in our environment for safe consumption [4].

We report two adult male patients who presented at the Obafemi Awolowo University Teaching Hospitals Complex, Ile-Ife, Osun state, South-West, Nigeria. They presented with predominantly central nervous manifestation of multiple convulsions following ingestion of undercooked bitter yam. The cases reported here are unique because of their rarity and favourable response to supportive treatment, and also they serve to add to the existing literature on this potentially reversible cause of acute encephalopathy.

## 2. Case 1

A 65-year old male cocoa farmer who was seen in the emergency room with multiple convulsions and loss of consciousness of 24-h duration. The convulsions started about 30 min after he ingested undercooked bitter yam which was harvested from his farm in Ipetumodu, a town about 30 min from Ile-Ife. He was reported to have cooked the yam for about 40 min instead of the usual 1 hour because he was very hungry. Other family members do not eat the yam because of the bitter taste.

The convulsions were described as generalized tonic–clonic in nature with associated urinary incontinence. Each episode lasted about 2 minutes and occurred at an average of three episodes per hour with no recovery of consciousness in between. This was the first episode of seizures in his life. He had no history of alcohol or psychoactive substance use. He was initially taken to a primary health centre where he received multiple doses of intravenous diazepam up to 20 mg which failed to control the seizures, although the frequency reduced to one episode in 2 hours.

At presentation in the emergency room, he was unconscious with a Glasgow coma scale of 9/15 (E2, V2, M5). His pupils were 3-mm round and reactive to light and had global hypertonia and hyperreflexia. He had a pulse rate of 90 beats per minute and a blood pressure of 120/80 mmHg. He was tachypnoeic with an oxygen saturation of 96% on intranasal oxygen and had widespread transmitted sounds on chest auscultation.

**Figure 1:**
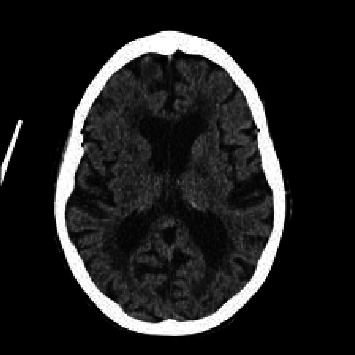
A noncontrast axial cranial CT image of the patient showing dilated sulcal spaces consistent with age-related brain atrophy and no other abnormalities.

Blood glucose was normal (4.6 mmol/L). Other baseline investigations: full blood count, serum electrolytes (sodium, potassium, ionized calcium, magnesium, chloride and bicarbonate), urea and creatinine, liver function tests, retroviral and hepatitis B/C screening were all within normal limits. An urgent noncontrast cranial computed tomography scan was also done to rule out a haemorrhage which was unremarkable, see Figure 1. A leftover sample of the bitter yam was inspected and confirmed by a botanist to be Dioscorea dumetorum; however, toxicologic testing of the yam or serum dioscorine levels of the patient could not be done due to unavailability of testing facilities in our centre.

A diagnosis of generalized convulsive status epilepticus secondary to bitter yam poisoning was made. Other diagnoses considered were haemorrhagic stroke, infectious encephalitis, hyperglycaemic emergency and intracranial space-occupying lesions. Following the persistence of the seizures despite two doses of intravenous diazepam given at the referring centre, he was commenced on the loading dose of IV phenytoin 900 mg (15 mg/kg) followed by maintenance doses 100 mg every 8 h as well as intravenous fluids.

He had no further seizures and regained full consciousness after 6 h on admission. Maintenance doses of IV phenytoin was continued for 48 h and subsequently stopped, and he was discharged without any deficits after 72 h on admission. Electroencephalogram done at the time of discharge was normal. He was followed up for 6 months at the Neurology clinic and had no further seizures.

## 3. Case 2

A 60-year old male electrician who presented at the emergency room on account of multiple convulsions of 8-h duration. He started feeling dizzy 1 hour after eating bitter yam which he bought from the local market in Ile-Ife. This is the first time he ate bitter yam and felt he should try it out. He was reported to have cooked it for about 30 min before eating.

Shortly after the onset of the dizziness, he started convulsing. Convulsions were described as generalized tonic–clonic with each episode lasting for less than 1 minute and followed by postictal sleep that lasted 20 min. He had an average of one episode of seizure per hour with full recovery of consciousness in between episodes. This is the first episode of seizure in his life. He has a background history of hypertension but well controlled on amlodipine 10 mg daily. He was initially rushed to a private facility where the seizures failed to resolve with three doses of intravenous diazepam with a total dose of 30 mg.

At presentation at the emergency room, he had 2 additional seizures each lasting about 3 min but was conscious in between seizure episodes. He had normally sized pupils which were reactive to light and had no cranial nerve deficit or cerebellar signs. He was tachycardic with a pulse rate of 102 beats per minute and a blood pressure of 112/78 mmHg. Oxygen saturation was 98% in room air with vesicular breath sounds.

**Figure 2:**
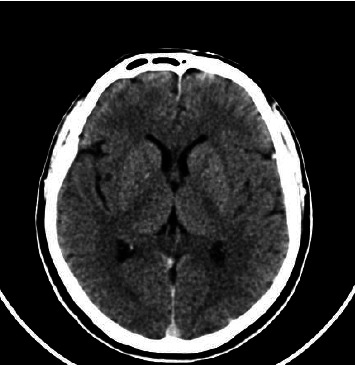
An axial noncontrast cranial CT image of the patient showing no abnormalities.

Random blood glucose was normal (6.4 mmol/L) as well as other baseline blood work-up (full blood count, electrolytes, urea and creatinine, liver function test, retroviral and hepatitis B/C screening) were all within normal limits. A noncontrast cranial computed tomography scan was also unremarkable, see Figure 2. There was no leftover sample of the bitter yam available for inspection, and serum dioscorine levels of the patient could not be measured at the centre due to unavailability of testing facilities.

A diagnosis of acute repetitive seizures secondary to bitter yam poisoning was made. Other diagnoses considered include haemorrhagic stroke, hyperglycaemic emergency and infectious encephalitis. Following persistence of the seizures after two doses of intravenous diazepam, he was also started on the loading dose of intravenous phenytoin 1050 mg (15 mg/kg) followed by maintenance doses 100 mg very 8 h as well as intravenous fluids.

He had no further seizures 3 h after admission. Phenytoin was discontinued after 48 h, and he was discharged on the 3rd day of admission with no residual deficits. Electroencephalogram done on outpatient basis was also normal. He was also followed up for 6 months at the Neurology outpatient clinic with no further complaints.

## 4. Discussion

These cases highlight ingestion of undercooked bitter yam as an important, potentially fatal but underreported cause of toxic encephalopathy, especially in regions like ours where yam is a popular delicacy. However our inability to do toxicological studies on the yam samples as well as serum dioscorine levels of the patients was a limitation.

Yam is a popular staple food in Nigeria and most regions of the world [5]. The bitter yam species Dioscorea dumetorum is found mostly in our locality. In most regions of Nigeria, bitter yam is popular among locals, and it is believed to have several health benefits [1, 4, 6]. It generally takes a minimum of 1 hour to cook under intense heat as a result of difficulty in separating the yam tissue cells. This is due to deposition of a tough high molecular weight lignin-like material in the cell wall [5]. Processing is usually done by soaking in water for long hours and boiling till fully cooked [3]. These methods have been shown to significantly decrease dioscorine levels [7].

Bitter yam can also be toxic with multiple organ system involvement if eaten raw or undercooked [2]. The toxic components of bitter yam include a toxic alkaloid dioscorine, as well as histamines [2, 3]. Dioscorine is responsible for the neurological manifestations of toxicity such as convulsions [2], while histamine is responsible for allergic manifestations such as pruritus, rashes and sometimes diarrhoea [3]. The exact mechanism of inducing convulsions by dioscorine is unclear. However, it is thought to be from its action on nicotinic acetylcholine receptors and antagonism of GABA and NMDA receptors [8].

There is a paucity of reported cases of bitter yam poisoning in our environment. However, in Thailand, Joob and Wiwanitkit [3] reported a 37-year-old female who presented with itching following ingestion of Asciatic bitter yam. Kang and Heo [9] also reported a case of acute kidney injury with toxic encephalopathy in a 51-year-old man who drank juice squeezed from the raw tubers of *Dioscorea quinqueloba*. Likewise in Ido-Ekiti, South-West Nigeria, Omefe et al. [10] in 2021 reported 3 siblings who developed convulsions following ingestion of undercooked bitter yam.

These referenced cases are similar to the two index cases we reported, in that there is a temporal association with ingestion of bitter yam species and evolution of symptoms, response to supportive treatment and full recovery. Intravenous phenytoin was used and was effective in controlling the seizures in the two cases reported. Intravenous phenytoin is one of the effective second line antiseizure medications approved for use in status epilepticus alongside intravenous sodium valproate and levetiracetam [11]. However, intravenous phenytoin is more readily available and affordable in our locality.

## 5. Conclusion

The above cases highlight toxicity of undercooked bitter yam notwithstanding its nutritive, potential medical properties and its widespread appeal in our locality. There is, therefore, the need for public health education in our communities on preventive measures as well as creating more awareness for healthcare practitioners in recognizing this rare cause of toxic encephalopathy.

## Data Availability

The datasets used are available from the corresponding author upon reasonable request.

## Patient Perspective

Patients' perspectives were mostly verbal and could not be documented, but they were very satisfied with the outcome.
